# The relation between children’s constructive play activities, spatial ability, and mathematical word problem-solving performance: a mediation analysis in sixth-grade students

**DOI:** 10.3389/fpsyg.2014.00782

**Published:** 2014-07-17

**Authors:** Meike Oostermeijer, Anton J. H. Boonen, Jelle Jolles

**Affiliations:** Faculty of Psychology and Education, Department of Educational Neuroscience, VU UniversityAmsterdam, Netherlands

**Keywords:** constructive play, spatial ability, mental rotation, mathematical word problem-solving performance, elementary school

## Abstract

The scientific literature shows that constructive play activities are positively related to children’s spatial ability. Likewise, a close positive relation is found between spatial ability and mathematical word problem-solving performances. The relation between children’s constructive play and their performance on mathematical word problems is, however, not reported yet. The aim of the present study was to investigate whether spatial ability acted as a mediator in the relation between constructive play and mathematical word problem-solving performance in 128 sixth-grade elementary school children. This mediating role of spatial ability was tested by utilizing the current mediation approaches suggested by Preacher and Hayes (2008). Results showed that 38.16% of the variance in mathematical word problem-solving performance is explained by children’s constructive play activities and spatial ability. More specifically, spatial ability acted as a partial mediator, explaining 31.58% of the relation between constructive play and mathematical word problem-solving performance.

## INTRODUCTION

In its home and school environment, almost every child is involved in playing with Legos, blocks, and jigsaw puzzles. The term *constructive play*, which has a central role in this study, is often used to categorize these play activities. Constructive play generally involves the manipulation, construction, and motion of objects in space (i.e., rotating, Mitchell, 1973; Pomerleau et al., 1990; Caldera et al., 1999). The aim of the present study is to examine the link between children’s constructive play activities and two interrelated factors, namely spatial ability and mathematical word problem-solving performance. Although a positive relation between constructive play and spatial ability is reported by several authors (e.g., Bjorklund and Douglas-Brown, 2008; Levine et al., 2012) as well as a positive relation between spatial ability and mathematical word problem-solving performance (Blatto-Vallee et al., 2007; Beentjes, 2008; Casey et al., 2008), a relation between constructive play and mathematical word problem-solving performance is barely investigated. A possible reason for this absence is that spatial ability acts as a mediator in the relation between children’s constructive play activities and their performances on mathematical word problems. The present study is primarily focused on testing this mediating role of spatial ability.

### THE RELATION BETWEEN CONSTRUCTIVE PLAY AND SPATIAL ABILITY

The majority of the studies that examined constructive play has focused on its relation with (the development of) spatial ability (e.g., Grimshaw et al., 2002; Bjorklund and Douglas-Brown, 2008; Levine et al., 2012). Spatial ability involves the ability to represent, modify, generate, and recall symbolic, non-linguistic information (Linn and Petersen, 1985; Tracy, 1987; Hegarty and Waller, 2005). Generally, three categories of spatial ability are distinguished in the literature, namely spatial perception, spatial visualization, and mental rotation (Linn and Petersen, 1985; Hegarty and Waller, 2005). Spatial perception involves determining spatial relationships with respect to the orientation of one’s own body, in spite of distracting information. Spatial visualization is commonly associated with tasks that involve complicated, multistep manipulations of spatially presented information. Mental rotation includes the ability to mentally remember and subsequently rotate an object in the space (Linn and Petersen, 1985; Hegarty and Waller, 2005). Numerous studies have demonstrated that constructive play activities contribute to the development of spatial ability, in specific mental rotation (Tracy, 1987; Brosnan, 1998; Caldera et al., 1999; Wolfgang et al., 2001; Grimshaw et al., 2002; Bjorklund and Douglas-Brown, 2008; Levine et al., 2012). In the present study, spatial ability is, therefore, referred to as the performance on mental rotation tasks.

According to the scientific literature, constructive play activities like Legos, blocks, and jigsaw puzzles exert the most influence on spatial ability (Mitchell, 1973; Pomerleau et al., 1990; Caldera et al., 1999; Levine et al., 2012). For example, evidence shows that the more children play with Legos, the more they improve in their spatial skills (Brosnan, 1998; Wolfgang et al., 2003). Besides playing with Legos, also block play has shown a positive relation with children’s spatial ability (Sprafkin et al., 1983; Tracy, 1987; Caldera et al., 1999). Preschool children that are more interested in block play and reproducing complex block models perform better on spatial ability tasks. Also jigsaw puzzles are examined in relation with spatial ability. Recent research of Levine et al. (2012) has revealed that the frequency of playing with jigsaw puzzles contributed to the development of spatial ability. Jigsaw puzzles appear to appeal to both the mental and physical rotation of the pieces to fit them into different places.

### THE RELATION BETWEEN SPATIAL ABILITY AND MATHEMATICAL WORD PROBLEM SOLVING

Besides the positive relation between constructive play and children’s spatial ability, a positive relation between spatial ability and mathematical ability, particularly mathematical word problem solving, is also reported in several studies (Guay and McDaniel, 1977; Lean and Clements, 1981; Tracy, 1987; Casey et al., 1992; Hegarty and Kozhevnikov, 1999; Kozhevnikov et al., 2002; Blatto-Vallee et al., 2007; Beentjes, 2008). Blatto-Vallee et al. (2007) showed, for example, that spatial ability explained almost 20% of unique variance in mathematical word problem-solving performance. Casey et al.(1997, 2001, 2008) reported that the direct role of spatial ability in mathematical word problem solving lies in performing the actual mathematical operations and numerical reasoning. Other studies have shown the importance of spatial ability in the production of visual-schematic representations (e.g., Hegarty and Kozhevnikov, 1999; Van Garderen, 2006; Krawec, 2010). In order to facilitate the understanding of the text base of a mathematical word problem, one has to make a coherent visual representation of the essential information of the problem. These visual representations include the spatial relations between solution-relevant elements of the word problem text (e.g., Hegarty and Kozhevnikov, 1999; Van Garderen and Montague, 2003; Thevenot and Oakhill, 2006; Van Garderen, 2006; Thevenot, 2010). To be able to make these types of representations, spatial ability is needed. So, children with good spatial skills are better able to make visual-schematic representations than children with poor spatial skills (e.g., Hegarty and Kozhevnikov, 1999; Van Garderen and Montague, 2003; Van Garderen, 2006; Krawec, 2010). The production of visual-schematic representations is found to be positively related to the performance on mathematical word problems (Van Garderen and Montague, 2003; Van Garderen, 2006; Krawec, 2010).

### THE PRESENT STUDY

In summary, the scientific literature reports a positive relation between children’s constructive play activities and spatial ability. Spatial ability increases as children engage more in playing with Legos, blocks, and jigsaw puzzles (Linn and Petersen, 1985; Tracy, 1987; Hegarty and Waller, 2005). Moreover, the positive relation between spatial ability and mathematical word problem-solving performance is also commonly investigated (Casey et al., 1997, 2001, 2008; Hegarty and Kozhevnikov, 1999). A relation between constructive play and mathematical word problem-solving performance is, however, not reported yet. A limited amount of studies have shown a positive relation between constructive play and more general math skills (Serbin and Connor, 1979; Caruso, 1993; Wolfgang et al., 2001). For example, the studies of Wolfgang et al. (2001) and Beentjes (2008) revealed that block play among preschoolers was a predictor of later school achievement in mathematics, when controlled for IQ and gender. All these studies did, however, not have a focus on mathematical word problem solving in particular. To our knowledge, this is one of the first studies that investigate the relation between constructive play and mathematical word problem-solving performance with spatial ability serving as a mediator. According to the statistical literature, a mediator explains the relation between the independent and the dependent variable. Rather than hypothesizing a direct causal relationship between the independent variable and the dependent variable, a mediational model hypothesizes that the independent variable causes the mediator variable, which in turn causes the dependent variable (for more information, see Shrout and Bolger, 2002; Preacher and Hayes, 2008; Hayes, 2009).

**FIGURE 1:**
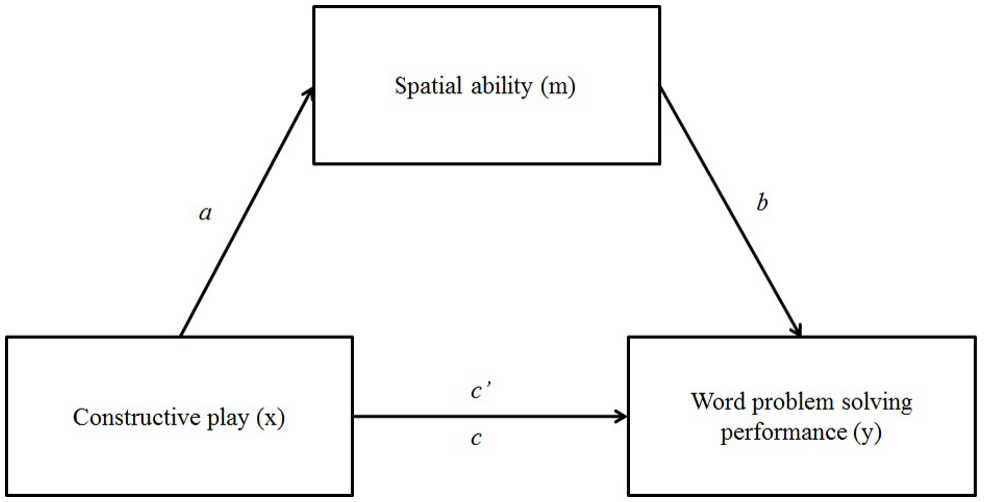
**Hypothesized mediation model including the independent variable (i.e., constructive play, *x*), mediator (i.e., spatial ability, *m*), and dependent variable (i.e., word problem solving performance, *y*)**.

The mediating role of spatial ability in the relation between constructive play and mathematical word problem-solving performance is reflected in the hypothetical (mediation) model reported in **Figure 1**.

As studies have demonstrated that there is a difference in the extent in which boys and girls are engaged in constructive play activities (see e.g., Serbin and Connor, 1979; Tracy, 1987; Scholten, 2008), sex is added as a covariate to the mediation model.

## MATERIALS AND METHODS

### SAMPLE

This study contained data from 128 Dutch sixth-grade children (64 boys, *M*_age_ = 11.73 years, SD_age_ = 0.43 years and 64 girls, *M*_age_ = 11.72 years, SD_age_ = 0.39 years) from eight elementary schools in The Netherlands. Parents/caretakers provided written informed consent based on printed information about the purpose of the study.

### INSTRUMENTS AND MEASUREMENT PROCEDURE

Children’s mathematical word problem-solving performance and spatial ability were administered by three trained independent research assistants in a session of approximately 25 min. Constructive play was examined with a questionnaire filled out by one of the parents/caretakers.

#### Mathematical word problem-solving performance

Mathematical word problem-solving performance was examined with the Mathematical Processing Instrument (MPI), which was first translated to Dutch. The MPI consisted of 14 mathematical word problems based on previous studies (Hegarty and Kozhevnikov, 1999; Van Garderen and Montague, 2003, see Appendix A). The internal consistency (Cronbach’s α) of this instrument, measured in American participants, is 0.78 (Hegarty and Kozhevnikov, 1999). The Cronbach’s α of the MPI in this study was 0.72. The word problems were printed on cards and presented in four different orders. All problems were read out loud to the children to control for differences in decoding skill. Furthermore, children were allowed to solve each word problem within a maximum of 3 min and during this time the experimenter did not speak to the child. To be sure those children had enough time to solve the mathematical word problems, a pilot study was conducted with five sixth-grade students. The results of the pilot study showed that every child was able to solve each of the 14 items of the MPI within the required 3 min. The number of mathematical word problems solved correctly was used as the dependent variable in the analyses.

#### Constructive play

In order to determine to what extent children show constructive play behavior, a short questionnaire was forwarded to their parents/caretakers. They were asked to indicate on a 4-point Likert scale (1 = never, 4 = often) to what extent their child has undertaken the, for this study, representative constructive play activities (i.e., playing with Legos, blocks, and jigsaw puzzles). The internal consistency of this questionnaire was sufficient (Cronbach’s α = 0.71). A sum score was created by adding the scores on the three, for this study representative, constructive play activities. The higher is the sum score, the more the student is involved in constructive play activities. The sum score was added as the independent variable in the analyses.

#### Spatial ability

**FIGURE 2:**
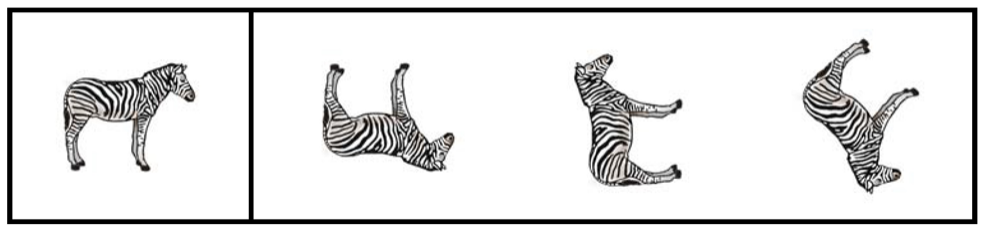
**The picture rotation task (based on Quaiser-Pohl, 2003)**.

The picture rotation task (Quaiser-Pohl, 2003) is a standardized task that was used to measure mental rotation. In the picture rotation task, children were asked to rotate a non-manipulated picture of an animal at the left of a vertical line. The three pictures at the right of the vertical line showed the rotated and/or mirrored image of that same animal. One of these three pictures was only rotated; two of these pictures were both rotated and mirrored. Children had to decide which of the three pictures was only rotated. Children had 1.5 min to finish this task. The internal consistency of this measure in the present study was high (Cronbach’s α = 0.93). **Figure 2** shows one of the 30 test items of the picture rotation task. The accuracy on this task was used as the mediator in the analyses.

### DATA ANALYSIS

The mediating effect of constructive play on word problem-solving performance via spatial ability was tested using bootstrap methods (Shrout and Bolger, 2002; Hayes, 2009). Bootstrap method has been validated in the literature and is preferred over other methods in assessing the existence of mediation among variables. Preference based on the fact that other methods for testing indirect effects assume a standard normal distribution when calculating the *p*-value for the indirect effect, whereas bootstrapping does not assume normality of the sampling distribution. In addition, bootstrap method repeatedly samples from the dataset, estimating the indirect effect with each resampled dataset. This process is repeated thousands of times, producing bias-corrected accelerated confidence intervals for the indirect effect (Preacher and Hayes, 2008).

## RESULTS

### DESCRIPTIVE STATISTICS

**Table 1** presents the correlations between, and the means and standard deviations of, the four measures of this study. The table shows that the correlations among the constructive play, spatial ability and word problem-solving performance are moderate to strong. No significant correlation is found among sex and the three key measures of this study.

**Table 1:** Intercorrelations, means, standard deviations, and ranges of the measures of this study.

	1	2	3	4	*N*	*M*	SD	Range
1. Constructive play	–				73	7.22	2.22	9.00
2. Spatial ability	0.25*	–			128	13.25	7.45	27.00
3. Word problem-solving performance	0.35**	0.55**	–		128	6.68	2.87	14.00
4. Sex	-0.16	-0.14	-0.16	–	128	–	–	–

*p< 0.05, **p < 0.001.

### INVESTIGATING THE MEDIATING ROLE OF SPATIAL ABILITY

Mediation was tested by regressing the dependent variable (i.e., word problem-solving performance) on spatial ability in the presence of constructive play and sex. Analyses utilizing the bootstrap method (5000 bootstrap samples were used) confirmed the existence of a mediation effect of constructive play on word problem-solving performance via spatial ability (see **Table 2**). However, the results showed that there is a partial, but not complete mediation, because the measured effect between constructive play and word problem-solving performance is not zero upon fixing the mediator variable (i.e., spatial ability, Preacher and Hayes, 2008). The value of the indirect effect of spatial ability can be calculated as follows:

**Table 2:** Parameter estimates of the model examining the mediation role of spatial ability in the relation between constructive play and word problem-solving performance.

Model	Estimate	SE	*p*	CI (lower)	CI (upper)
**Model without mediator**
Intercept	4.57	1.11	<0.01	2.36	6.78
CP→WPS (*c*)	0.38	0.14	<0.01	0.11	0.66
Sex→WPS	-1.44	0.60	<0.05	-2.63	-0.24
*R^2^* (*y*, *x*)	0.19	–	–	–	–
**Model with mediator**
Intercept	2.86	1.04	<.01	0.78	4.94
*Model 1: SP as dependent variable*				
CP→SP (*a*)	0.75	0.41	<0.05	-0.06	1.56
Sex→SP	-3.33	1.79	0.07	-6.90	0.25
*Model 2: WPS as dependent variable*
SP→WPS (*b*)	0.16	0.04	<0.001	0.09	0.23
CP→WPS (*c’*)	0.26	0.12	<0.05	0.02	0.51
Sex→WPS	-0.89	0.54	0.10	-1.97	0.18
Indirect effects (*a* x *b*)	0.12	0.08	<0.05	0.128	0.27
*R^2^* (*m*, *x*)	0.10	–	–	–	–
*R*^2^ (*y*, *m*, *x*)	0.38	–	–	–	–

**FIGURE 3:**
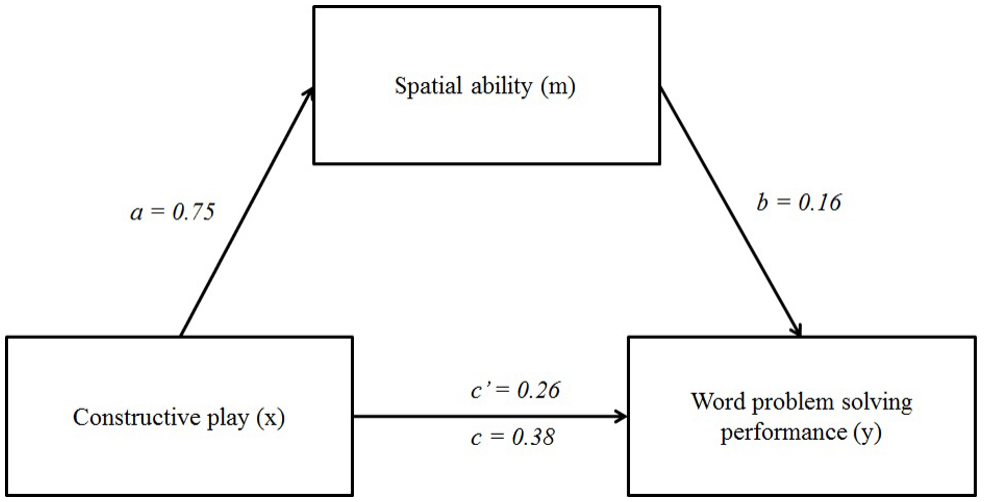
**Results of the mediation analysis (*N* = 128).** Sex was included in the equations as a statistical control but is not presented for reasons of clarity.

*Regression weights *a*, *b*, *c*, and *c’* are illustrated in **Figure 3**. *R^2^* (*y*, *x*) is the proportion of variance in *y* explained by *x*, *R^2^* (*m*, *x*) is the proportion of variance in *m* explained by *x* and *m*. the 95% CI for *a* × *b* is obtained by the bias-corrected bootstrap wit 5000 resamples. CP (constructive play) is the independent variable (*x*), SP (spatial ability) is the mediator (*m*), and WPS (word problem-solving performance) is the outcome (*y*). CI (lower = lower bound of 95% confidence interval; CI (upper) = upper bound.*

*B*_indirect_ = *B*_(a)_^∗^*B*_(b)_ = 0.75 × 0.16 = 0.12, and

*B*_indirect_/*B*_total_ = 0.12/0.38 = 0.3158.

Thus, spatial ability explained 31.58% of the relation between constructive play and students’ mathematical word problem-solving performance. The absence of a zero in the confidence interval for the indirect pathways indicated that the indirect effect was significantly different from zero at *p* < 0.05, two-tailed.

The complete model (including constructive play, spatial ability, and sex) explained 38.16% of the variance in students word problem-solving performance (*R*^2^ = 0.38), which is a large effect (Green and Salkind, 2008; Fairchild et al., 2009).

## DISCUSSION

The purpose of the present study was to investigate if spatial ability acts as a mediator in the relation between constructive play and mathematical word problem-solving performance in sixth-grade elementary school children. To our knowledge, this is one of the first studies that examined the mediating role of spatial ability in this particular relation. In previous studies, relations between constructive play and spatial ability (e.g., Brosnan, 1998; Bjorklund and Douglas-Brown, 2008), and between spatial ability and mathematical word problem-solving performance (e.g., Hegarty and Kozhevnikov, 1999; Van Garderen and Montague, 2003; Blatto-Vallee et al., 2007) are reported. The relation between constructive play and mathematical word problem-solving performance has, however, not been established yet.

The results of this study showed that spatial ability acted as a partial mediator in the relation between constructive play and children’s mathematical word problem-solving performance. This implies that children who were frequently engaged in constructive play in their past have better spatial skills and, as a result, show a higher performance on mathematical word problems. The variables in this study (i.e., constructive play, spatial ability, and sex) explained 38.16% of the variance in performance on solving mathematical word problems. Furthermore, 31.58% of the relation between constructive play and mathematical word problem-solving performance is explained by spatial ability.

Note that the findings of this study support the assessment of a mediating effect based on current recommendations using bootstrap approaches (Shrout and Bolger, 2002; Hayes, 2009)^1^.

### LIMITATIONS

Three limitations of the study should be mentioned. The first limitation of this study included the fact that only one task was used in the analyses to measure spatial ability (i.e., mental rotation). Ideally, method triangulation should be applied. The use of more tasks allows a more reliable measurement of the construct “spatial ability” and reduces the chance of possible measurement errors (Woolderink, 2009). The second limitation pertains to the correlational nature of the data, which made it impossible to draw conclusions about any causal relationships among constructive play, spatial ability, and mathematical word problem-solving performance. The results of this study only showed that these variables were associated with each other. Future experimental studies in which the variables will be manipulated should make it possible to draw stronger conclusions concerning causal relationships between the aspects which are important in mathematical word problem solving. The last limitation covers the way in which the constructive play activities of the children were administered. In the present study, a third party (i.e., the parents), filled out the questionnaires regarding the extent to which children show constructive play behavior. Although parents were able to provide a reliable image of the constructive play activities in which their children are/were involved, in future studies it would be even more reliable to directly observe these play activities.

### IMPLICATIONS AND DIRECTIONS FOR FUTURE RESEARCH

The present study contributed to the increasing amount of scientific literature regarding the processes that are involved in learning mathematics, particularly mathematical word problem solving. An interesting focus of future research is to investigate the existence of individual differences in the specific relations between the three key variables of this study (i.e., constructive play, spatial ability, and mathematical word problem-solving performance). Although not supported by the results of the present study, several authors have demonstrated that boys and girls differ in the extent in which they engage in constructive play (Serbin and Connor, 1979; Tracy, 1987; Scholten, 2008). That is, boys tend to play more with so-called masculine or constructive toys, like Legos and blocks, than girls (Serbin and Connor, 1979; Tracy, 1987). Because the scientific literature gives no clear indications that sex differences exist in spatial ability (e.g., McGee, 1979; Voyer et al., 1995), examining the mediating role of spatial ability for both boys and girls separately might be an interesting topic for follow-up studies.

The results of this study also have a strong practical relevance. Parents/caretakers should be aware of the importance of constructive play activities in childhood. According to the findings of this study, activities like playing with Legos, blocks, and jigsaw puzzles, are positively related to students’ spatial skills, which, in turn, is positively related to their performance on mathematical word problems. Parents/caretakers should, therefore, create opportunities to play with constructive toys. Also elementary school teachers should provide constructive learning material to their children and stimulate to use it by giving them appropriate instruction. Finally, this research accentuated the importance of spatial ability in mathematical word problem-solving performance. In line with previous research (e.g., Hegarty and Kozhevnikov, 1999; Van Garderen, 2006), spatial ability was found to play a key role in solving mathematical word problems, especially in the production of visual-schematic representations. The training of spatial skills and the development of visual-schematic representations should, therefore, have a prominent role in word problem-solving instruction of primary school mathematics education.

## Conflict of Interest Statement

The authors declare that the research was conducted in the absence of any commercial or financial relationships that could be construed as a potential conflict of interest.

## Footnotes

1 This assessment of mediation is also support by the statistical approach that Baron and Kenny (1986) used in their research.

## APPENDIX

The mathematical word problems on the MPI (Hegarty and Kozhevnikov, 1999):

1. At each of the two ends of a straight path, a man planted a tree and then every 5 m along the path he planted another tree. The length of the path is 15 m. How many trees were planted?
2. On one side of a scale, there is a l kg weight and half a brick. On the other side, there is one full brick. The scale is balanced. What is the weight of the brick?
3. A balloon first rose 200 m from the ground, then moved 100 m to the east, then dropped 100 m. It then traveled 50 m to the east, and finally dropped straight to the ground. How far was the balloon from its original starting point?
4. In an athletics race, Jim is 4 m ahead of Tom and Peter is 3 m behind Jim. How far is Peter ahead of Tom?
5. A square (A) has an area of 1 m^2^. Another square (B) has sides twice as long. What is the area of square B?
6. From a long stick of wood, a man cut 6 short sticks, each 2 feet long. He then found he had a piece of 1 foot long left over. Find the length of the original stick.
7. The area of a rectangular field is 60 m^2^. If its length is 10 m, how far would you have traveled if you walked the whole way around the field?
8. Jack, Paul, and Brian all have birthdays on 1 January, but Jack is 1 year older than Paul and Jack is 3 years younger than Brian. If Brian is 10 years old, how old is Paul?
9. The diameter of a tin of peaches is 10 cm. How many tins will fit in a box 30 cm × 40 cm (one layer only)?
10. Four young trees were set out in a row 10 m apart. A well was situated beside the last tree. A bucket of water is needed to water two trees. How far would a gardener have to walk altogether if he had to water the four trees using only one bucket?
11. A hitchhiker set out on a journey of 60 miles. He walked the first 5 miles and then got a lift from a lorry driver. When the driver dropped him, he still had half of his journey to travel. How far had he traveled in the lorry?
12. How many picture frames 6 cm long and 4 cm wide can be made from a piece of framing 200 cm long?
13. On one side of a scale, there are three pots of jam and a 100 g weight. On the other side, there are a 200 g and a 500 g weight. The scale is balanced. What is the weight of a pot of jam?
14. A ship was sailing North-West. It made a turn of 90^∘^ to the right. An hour later it made a turn of 45^∘^ to the left. In what direction was it then traveling?
